# Successful Establishment of an Orthotopic Hepatoblastoma In Vivo Model in NOD/LtSz-scid IL2Rγnull Mice

**DOI:** 10.1371/journal.pone.0023419

**Published:** 2011-08-10

**Authors:** Verena Ellerkamp, Sorin Armeanu-Ebinger, Julia Wenz, Steven W. Warmann, Juergen Schäfer, Peter Ruck, Joerg Fuchs

**Affiliations:** 1 Department of Pediatric Surgery, Children's University Hospital Tuebingen, Tuebingen, Germany; 2 Department of Pediatric Radiology, Children's University Hospital Tuebingen, Tuebingen, Germany; 3 Institute for Pathology Leonberg, Leonberg, Germany; Université Pierre et Marie Curie, France

## Abstract

Investigation of hepatoblastoma in experimental conditions contributes relevantly to a detailed understanding of tumor biology and the investigation of new treatment approaches. Most systematical analyses currently use subcutaneous xenografts. We established a reproducible intrahepatic model with the hepatoblastoma-cell lines HuH6 and HepT1. The cells were stably transfected with a plasmid vector encoding for Gaussia luciferase. HuH6 and HepT1 were injected intrasplenically in NOD/LtSz-scid IL2Rγnull mice. Mice were splenectomized in order to avoid intrasplenical tumor growth. Multifocal intrahepatic tumor growth was observed in 85% (11/13) of HuH6 tumors and 55% (5/9) of HepT1 tumors. Serum Alpha-fetoprotein and Gaussia luciferase increased 5 weeks after tumor-cell inoculation. Tumors were detected by MRI at this time point. Immunhistochemical analysis such as vascularity (CD31), proliferation index (Ki-67), cytokeratin 7 and distribution of β -catenin in intrahepatic tumors were different to subcutaneous tumors. We established a reproducible xenograft model for intrahepatic hepatoblastoma growth with a high tumor incidence. Monitoring of tumor cell viability was optimized by measuring GLuc. This model enables further experimental investigations of HB in a more physiological milieu as emphasized by the β-catenin distribution.

## Introduction

Hepatoblastoma (HB) has an incidence of 0.6 per 100,000 children. Improved outcome was achieved through clinical trials such as the Childhood Liver Tumours Strategy Group (SIOPEL) and the study HB 89–99 of GPOH. In standard risk HB the 3-year survival is 91% and the 3-year event-free survival is 73% after preoperative chemotherapy [1]. Even reduced chemotherapeutic regimen with cisplatin monotherapy was recently proven to be as effective as cisplatin-doxorubicin combination in children with standard risk tumors [2]. High risk hepatoblastoma, defined as tumor growth in all liver sections (PRETEXT-IV), vascular invasion, intra-abdominal extrahepatic extension, metastatic disease, α-fetoprotein less than 100 ng/mL at diagnosis is reported to have an event-free 3-year-survival of 65% [3], needing more extensive therapeutic strategies. In contrast to the continuously improving results of the standard group no landmarks in cases of high risk HB are made [4]. Thus, within the scope of experimental therapeutic strategies alternative concepts are necessary.

Experimental anti-tumor therapies are frequently tested for efficacy in murine subcutaneous tumor models. Established HB cell lines that are currently used include the mixed HB cell line HuH6 and the embryonal HB cell line HepT1 [5]–[7]. For example studies of blockade of VEGF [8], or of modulation of multidrug resistance [9] are carried out using these models. Tumor tumor volume can be assessed by calculation of xenograft measurement [10]. However, the skin and subcutaneous tissue display a high inherent immunogenicity due to the abundant presence of antigen presenting cells and therefore subcutaneous tumors do not adequately mirror orthotopic tumour growth. Human tumors injected subcutaneously into mice usually grow as encapsulated masses with little evidence of local invasion or distant metastases [11]. Orthotopic injection of tumors frequently enhances their tumorigenic and/or malignant properties. For intrahepatic growth of hepatic tumors some methods are described. If the cells do not spread after intraperitoneal or intravenous injection they can be injected into the liver, portal vein or spleen. Formation of hepatic metastases subsequent to intrasplenic injection of tumor cells was first described by Leduc in 1959 [12]. In case of hepatoblastoma Schnater et al. first described an intrahepatic growth of intrasplenically injected HuH6 cell-lines in 25% of treated NMRI nu/nu mice [13]. These models were not used for testing of new therapeutic approaches, because of low tumor uptake. Our aim was to establish an improved model of orthotopic HB through alteration of essential parameters. The application of orthotopic tumor models in the liver implies a need for non-invasive techniques of tumor evaluation. For non-invasive tumor monitoring we used cell transfection with luciferase-genes for in vivo bioluminescence measurement and MRI.

## Results

### Induction of tumor growth

Intravenous (n = 4) or intraperitoneal (n = 4) cell injection did not lead to tumor growth. None of the mice developed measurable AFP or GLuc levels, and 7 weeks post injection there was no tumor visible at laparotomy.

**Figure 1:**
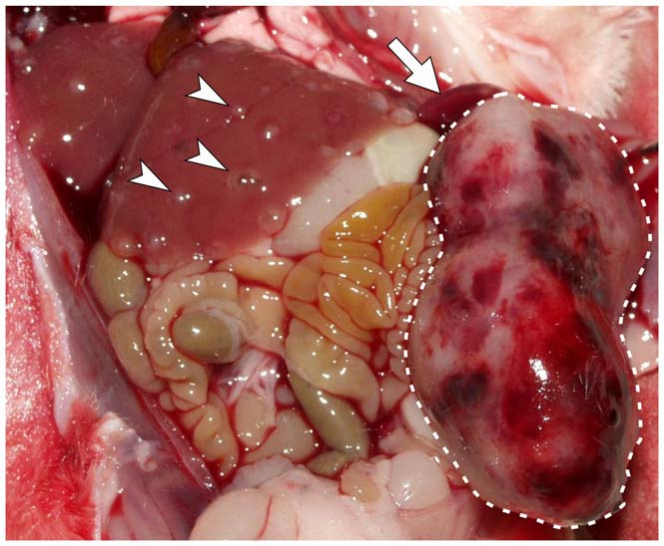
Tumor growth in the spleen with hepatic nodules. Without splenectomy, a bulky tumor mass develops in the spleen 4 weeks after intrasplenic HB cell injection (surrounded by broken line). The primary HuH6 derived tumor in the spleen compresses the bowels. Several disseminated intrahepatic nodules are present (arrow heads). The upper pole of the spleen is marked with an arrow.

**Figure 2:**
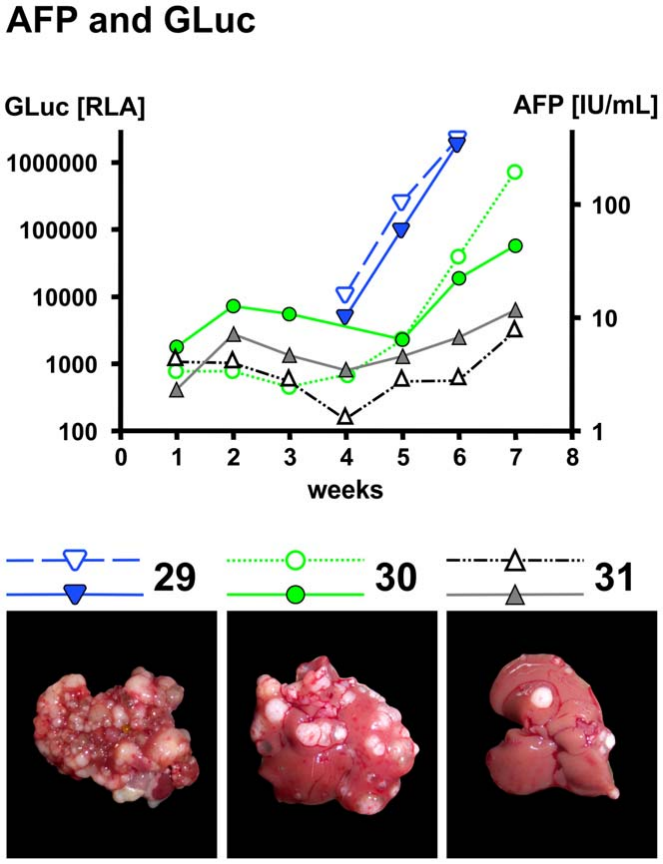
AFP and GLuc levels in relation to tumor size. Serum-AFP and GLuc weekly measurements shown in 3 representative mice. Following intrasplenic injection of HuH6 cells and splenectomy, AFP (IU/mL, broken lines with open signs) and GLuc activity (relative light units, lines with filed signs) begin to rise exponentially in all 3 depicted animals. Corresponding photographs of the liver at the base show intrahepatic tumor nodules, whose number is comparable to the level of the tumor marker detected in serum.

Intrasplenic injection of HuH6 cells without splenectomy (n = 6) led to HB growth in the spleen in all mice, and to small intrahepatic tumor nodules in 4 mice (Figure 1). At this time, the size of the splenic tumor led to bowel displacement and weight loss. The mean volume of the intrasplenic tumors was 1330 mm^3^ (960–1885). The volume of intrahepatic tumor nodules ranged from 0.5 mm^3^ to 4.5 mm^3^; due to the huge number of nodules, they were not countable. None of these mice showed detectable extrahepatic or pulmonary nodules. In this group AFP rose in the 3^rd^ week after cell injection, with the following mean scores: 2^nd^ week – 18.98 IU/mL (12.8–23.94); 3^rd^ week – 187.35 IU/mL (95–284.14); 4^th^ week – 526.88 IU/mL (331–1141.9). To avoid tumor growth at the site of injection (spleen), in following experiments a splenectomy was performed. This resulted in intrahepatic tumor growth in 83% mice injected with HuH6 cells. Intrahepatic HB growth of HepT1 cells was less frequent (Table 1). The intrahepatic tumor nodules varied in size and number: some mice had more than 20 nodules with a volume of 0.5–5 mm^3^, while other mice had less than 10 nodules with a volume of 60–400 mm^3^. Most nodules were cystic with high amounts of α-Fetoprotein (AFP) measurable in the fluid (1250 IU/l). Additional extrahepatic tumor nodules in the peritoneum, ovaries and abdominal wall were detected. In all mice with intrahepatic tumor growth of cells the AFP levels and GLuc increased 5 weeks after xenotransplantation. The mean values of AFP measurements at each time point did not differ significantly between HuH6 and HepT1 tumors (Mann-Whitney-test >0.05) and increased in parallel. The increase of Gaussia luciferase (GLuc) levels was more irregular at higher ranges within the group (Table 2). The increase of AFP and GLuc-activity were exponential and matched to the number of tumor nodules observed on the surface of the liver (Figure 2). As the number of the tumors was high and too numerous to count, the tumor load in the liver was analyzed only by the serum parameters AFP and GLuc.

**Table 1:** Incidence of intrahepatic tumor growth using HuH6 and HepT1 cells.

Splenectomy	Cell line	Intrahepatic tumor	Extrahepatic tumor	Intrasplenic tumor
−	HuH6	4/6 (67%)	0/6	6/6
+	HuH6	11/13 (83%)	2/13	
+	HepT1	5/10 (50%)	3/10	

The numbers indicate mice with tumors among all mice in the experiment. HuH6 cells showing a tendentially higher tumor incidence than HepT1 cells (Fisher test, contingency analysis p = 0.166).

**Table 2:** Time course of tumor cell markers for orthotopic HuH6 and HepT1 tumors.

	Week→	2	3	4	5	6	7
**AFP IU/ml**	**HuH6**	3.56 (2.97–4.14)	3.17 (2.46–4.87)	5,53 (1.31–16.16)	29,75 (6.61–105.89)	99,33 (34.41–380.69)	249,4 (193.45–251.39)
	**HepT1**	4.67 (3.89–4.96)	3.98 (3.57–5.22)	3.80 (3.46–4.89)	9.19 (7.25–13.4)	124,80 (35.28–239.64)	175,63 (157.12–194.65)
**GLuc RLA**	**HuH6**	13212 (6920–19290)	15330 (11990–18460)	16892 (12590–20500)	45507 (17300–113150)	564245 (21240–1945840)	108246 (25280–240860)
	**HepT1**	20435 (11930–22640)	45565 (28950–56880)	130735 (14.380–160060)	471582.5 (100220–968590)	4115462 (174260–12049330)	1881575 (1296890–2466260)

AFP and GLuc levels mean values (range) in week 2 to week 7 after tumor cell injection and splenectomy.

**Figure 3:**
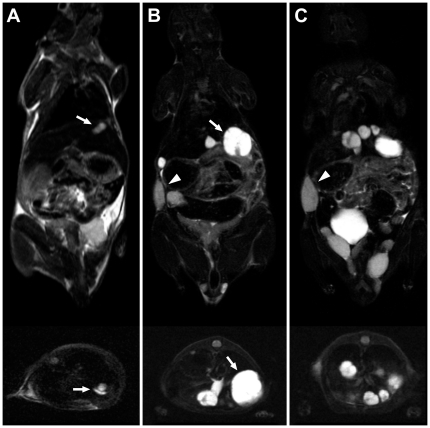
Exemplary MRI of HB tumors in mice. T2-weighed MRI; upper row, coronar; lower row, axial view. A: MRI 4 weeks after i.s. HuH6 cell injection, showing a small nodule in the left liver lobe (white arrow). B, C: the same mouse 6 weeks after injection. Several new nodules have developed and the first detectable nodule has grown (white arrow). A metastasis of the abdominal wall is detectable (arrow head). This mouse also shows ovarian tumor spread.

Weekly carried out MRI showed i.h. lesions as early as 5 weeks after HB cell injections. The smallest detectable lesions had a diameter of 1 mm. In T2 weighed sequences water- and fluid-containing tissues (e.g. vessels) were strongly demarcated from the surrounding tissue and had a bright appearance. T2-weighted imaging of a healthy murine liver demonstrated the dark healthy liver parenchyma. Tumor nodules were nonhomogeneous and hyperintense in T2 weighed sequences. Cystic regions as well as septa could be detected in larger lesions. In T1-weighted sequences, fat and fat containing tissue (e.g. bone marrow) appeared bright (hyperintense) and the structure of organs was clearly detectable. Tumor nodules were not visible in T1 weighed sequences (Figure 3).

### Histology and Immunohistochemistry

In subcutaneously growing tumors as well as intrahepatically growing tumors a histological picture of embryonal hepatoblastoma consisting mainly of sheets and trabeculae of epithelial tumor cells with eosinophilic cytoplasm and a high nucleocytoplasmic ratio was detected. Necrosis was seen to varying extents, with more necrosis dectectable in larger tumors and/or in subcutaneous tumors. Intrahepatically growing HB tumors showed more cystic areas compared to subcutaneous tumors. The cells of orthotopic tumors were of smaller size compared to s.c. cells.

**Figure 4:**
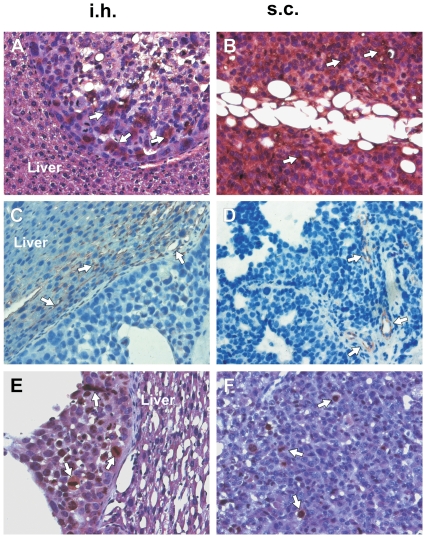
Immunohistological detection of AFP, CD31, and Ki-67 in HB tumors. Images show staining of intrahepatic (i.h.) tumors (left column), and of subcutaneous (s.c.) tumors (right column) grown from HuH6 cells for AFP (A,B), CD31 (C, D) and Ki-67 (E, F). Counterstaining with Haematoxilin and Eosin (A, B, D, E). Tumor areas with strong expression of the respective marker (brown staining) are highlighted with open arrows. The endothelium marked by CD31 is found in the tumor parenchyma of s.c. tumors, while in small i.h. tumors a high density of capillaries were found in the surrounding liver tissue. Original magnification of images: ×20.

**Figure 5:**
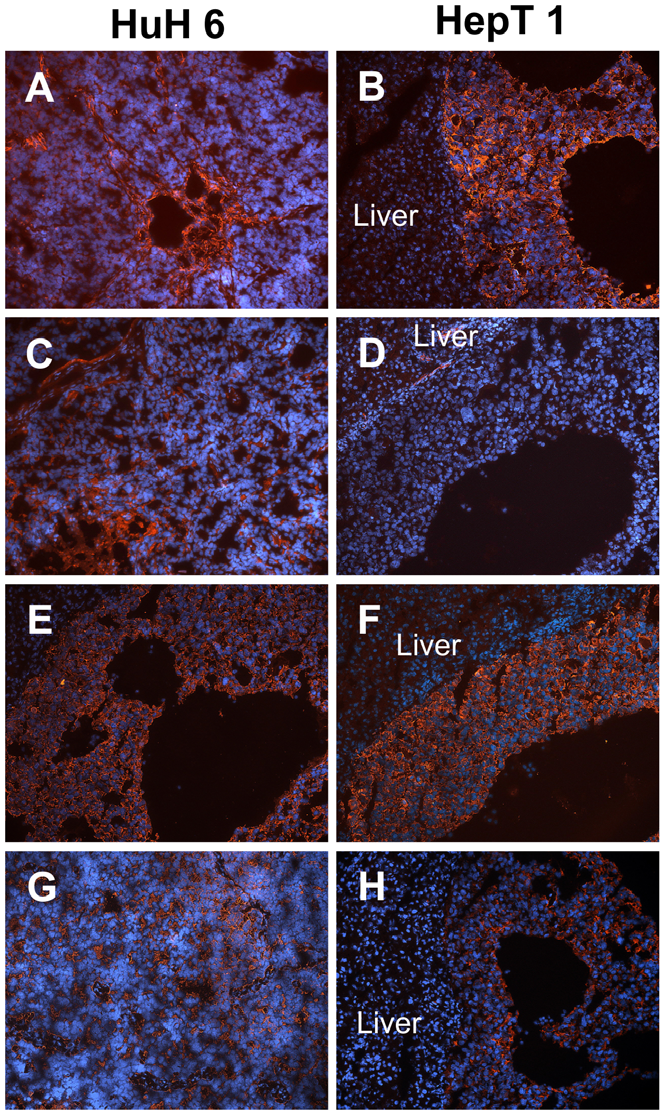
Expression of Vimentin, Cytokeratin 7, Cytokeratin AE and EpCam in i.h. tumors. Red fluorescence denotes expression of Vimentin (A,B), Cytokeratin 7 (C,D) and Cytokeratin AE (E,F) and EpCam (G,H) in i.h. tumors derived from HuH6 (left column) and in HepT1 (right column). Blue fluorescence depicts nuclei (DAPI staining). Original magnification of images: ×10.

**Figure 6:**
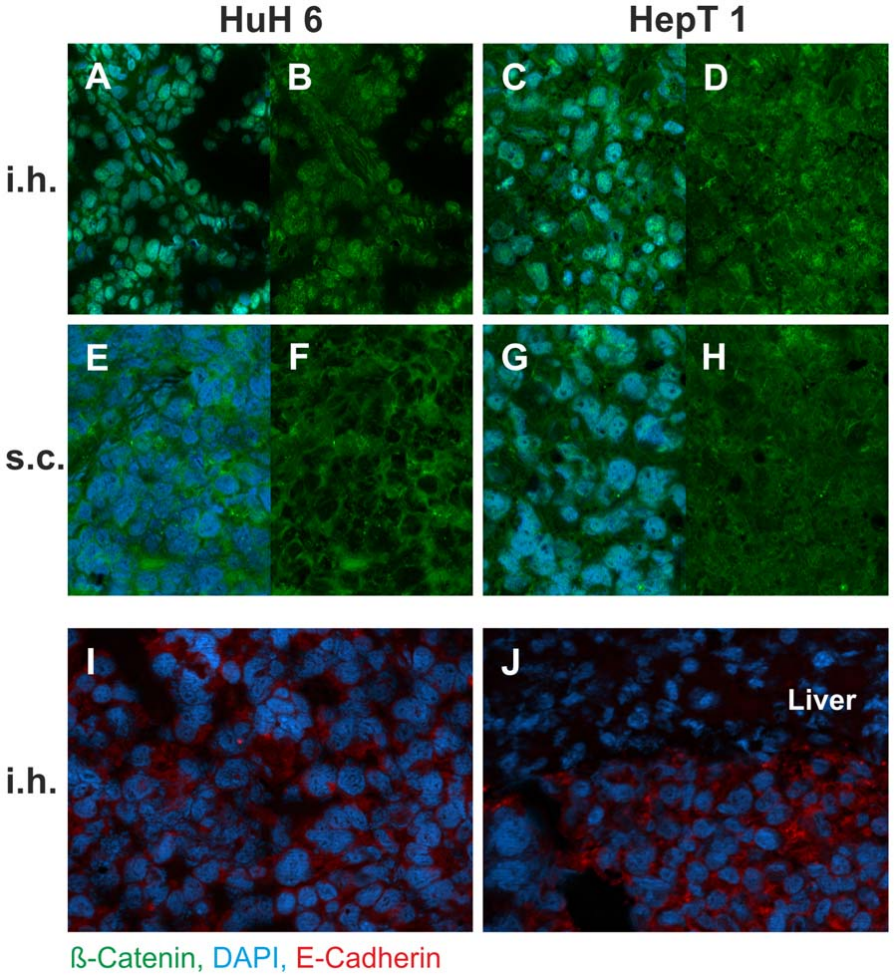
Expression of β-catenin and E-Cadherin in xenotransplantated HB Tumors. All tumor cells show green fluorescence staining for β-catenin in the cytoplasma and in the nucleus, while the specimens of intrahepatic xenografts show a notably higher β-catenin staining in the nuclei than the subcutaneous xenografts. This difference is more obvious in HuH6 tumors (A, B, E, F), but also detectable in HepT1 tumors (C, D, G, H). The staining for E-cadherin (I, J) show cytoplasmatic distribution in the tumor tissue from intrahepatically growing HuH6 (I) and HepT1 (J); there was no difference to s.c. xenografts (not shown). There is no E-Cadherin staining in the adjacent hepatic tissue (liver). A, C, E, G show specimen with DAPI nuclear counterstaining; B, D, F, H show the identical specimen without counterstaining. Original magnification of images: ×20 (A–H), ×40 (I,J).

Immunohistological findings of intrahepatic grown HB (after splenectomy) versus subcutaneously grown HB as well as HuH6 versus HepT1 tumors were compared. A summary of all findings is given in Table 3, and the representative findings are shown in Figures 4, 5, and 6. Notable differences were seen in the expression of Vimentin, which was stronger in all HepT1 tumors and CK7, being stronger in all HuH6 tumors.

**Table 3:** Immunohistochemical findings.

		Histology			Tumor proteins				Wnt-pathway, Adhesion	
Cell line	HB growth	Vasc. (CD31)	AFP	Proliferation(ki-67 LI%)	Vimentin	CK 7	CK AE	EpCam	β-catenin	E-cadherin
HuH 6	i.h.	(+)	+	48	‡	++	++	++	++ (n)	++
	s.c.	(+)	+	45	‡	+	++	++	++ (c)	++
Hep T1	i.h.	+	+	38	++	−	++	++	++ (n>c)	++
	s.c.	(+)	+	18	++	−	++	+	++ (c>n)	++

The results of immunostaining are symbolized by the positive rate of all stained cells: ++, over 80%; +, 80−40%; (+), 40−10%; –, less than 10% or negative; ‡ focally positive. n, nuclear; c, cytosplasmatic.

Staining with β-catenin showed a strong expression within all tumor specimens (Figure 6). There was an obvious difference in the β-catenin distribution pattern of intrahepatic and subcutaneous HB: in HuH6 intrahepatic tumors there was a strong immunoreactivity in the nucleus, barely in the cytoplasm, and none in the membrane. While most of the nuclei in the subcutaneous tumors showed no β-catenin, the cytoplasm did. This observation was also made in HepT1 tumors, but less marked than in HuH6 tumors. However, there was also some immunoreactivity in the nuclei of the subcutaneous tumors. β-catenin interacts with E-cadherin at the cell membrane and facilitates interaction of the cell adhesion molecule with the cytoskeleton protein α-actin. E-cadherin was similarly detectable in the cytoplasm of all intrahepatic and subcutaneous tumors without a difference between the HuH6 and HepT1 (Figure 6).

## Discussion

Models for hepatoblastoma are limited to three existing cell lines: HuH6 originating from an embryonal hepatoblastoma of a Japanese patient [6], HepT1 and HepT3 established from a poorly differentiated embryonal (HepT1) and a fetal and embryonal (HepT3) hepatoblastoma, respectively, as described [5]. For preclinical investigations the murine subcutaneous xenograft model was mainly used for the last 14 years [10]. HB tumors are usually growing subcutaneously without metastatic spreading, permitting an easy tumor volumetry during therapy [10]. Advantages of orthotopic tumor growth in experimental settings are described for entities other than HB [11]. For example, well established murine models exist for hepatocellular carcinoma, colorectal cancer, renal cell carcinoma, and pancreatic ductal carcinoma [14]–[17]. Concerning HB there have been some attempts to establish an orthotopic or alternate subcutaneous model: intrarenal injection with renal HB tumors in mice [8]; laparoscopic intraperitoneal seeded HB with peritoneal HB tumors in nude rats [18]; intrasplenic injection with intrahepatic HB tumors in mice [13]. While intrarenal and peritoneal HB xenografts are still lacking the orthotopic environment, the orthotopic HB model was impaired by a low incidence of intrahepatic tumor growth and the inability to track HB tumor volume and viability online.

To increase the incidence of mice with intrahepatic HB nodules we established a reliable model that can be used for further research of experimental treatments. One modification of existing models was the usage of another mouse strain with less immunologic competence. NOD/LtSz-scid IL2Rγnull (NSG) mice present deficiency of mature lymphocytes and NK cells [19], which is an important factor in allowing xenografts to grow. The superior engraftment of HB cells in NSG mice after injection of comparatively low numbers of cells may be a result of a lower number of Kupffer-cells and NK cells in these mice. Further in vitro studies will be carried out for a better understanding of these mechanisms.

Delivery of HuH6 cells into the spleen lead to small tumor nodules in the liver in 67% of the animals; however all treated mice developed large tumors in the spleen within 4 weeks. To prevent this large intrasplenic (ectopic) HB tumor, we resected the spleen shortly after HB cell injection. This resulted in multifocal intrahepatic HB xenografts in 85% of mice after HuH6 cell injection and in 50% of mice after HepT1 cell injection after 5 to 7 weeks, which means a higher incidence of i.h. nodules after splenectomy. Without splenectomy the incidence of i.h. HB nodules would probably increase with a longer time of observation. However due to a higher HB cell accumulation in the site of injection the intrasplenic tumor grows faster than the i.h. nodules. The intrasplenic tumor burden limited the observation period to 4 weeks. The high incidence of i.h. HB nodules after splenectomy suggests that an initial HB cell seeding via the splenic vein directly after tumor cell injection is responsible for an orthotopic tumor initiation and not a metastatic spreading from a primary splenic tumor. By avoiding a large tumor mass in the spleen through splenectomy, a better correlation of GLuc/AFP levels to tumor load within the liver is achieved. In treatment models, huge tumor masses in the spleen would hamper the monitoring of therapeutic effects.

The lower tumor incidence in HepT1 compared to HuH6 xenotransplanted mice has already been described [13]. This may be related to the lower proliferation rate of HepT1 cells compared to HuH6 cells: in cell culture the doubling time of HuH6 cells is twice as high as in HepT1 cells. Furthermore in HepT1 xenografts, the proliferation index marked by Ki-67 staining was inferior as compared to HuH6 cells.

The multifocal, solid-cystic growth pattern of i.h. HB differs from a more solid growth pattern in intrasplenic HB [13] or primary HB in men. Whether this is a result of the specific interaction between NSG liver-cells and HB cells remains unclear. This untypical growth pattern could perhaps present a certain limitation of this model. Reports on cystic HB especially in metastases and adult patients remain particularly noteworthy [20], [21].

In an orthotopic tumor model, non-invasive monitoring of the tumor growth and its vitality is critical. For monitoring of intraperitoneal HB xenografts, a photodynamic visualization of peritoneal HB xenografts in rats after intraperitoneal injection of 5-aminolevulinic acid is described. This method allows for monitoring of tumor size and vitality simultaneously, but does require laparoscopy [22]. In subcutaneous models the tumor bulk can easily be monitored by tumor volumetry [10], and AFP level measurements serve as marker of tumor vitality. In addition to AFP measurements, we measured GLuc expression. Transfection of HB cells with a plasmid encoding GLuc not only allows the measurement of another serum parameter increasing parallel to AFP, but also provides an observation of the tumor growth through in vivo bioluminescence: after injection of the GLuc substrate coelenterazine the photon counts can be acquired using a CCD camera [23].

A more exactly tumor volumetry is given by MRI avoiding the light absorption in the liver tissue. Anesthesia is needed for in vivo bioluminescence as well as for MRI. Due to its superior spatial resolution, we prefer the MRI for diagnostic imaging. The advantages of MRI in metastases detection compared to contrast enhanced CT are described in literature [24] and several protocols for early detection of hepatic metastases are available [25].

By comparing subcutaneously and intrahepatic xenografts we could specify the advantages of an orthotopic milieu as an experimental setting in HB. In the literature encapsulation and aberrant vascularisation are repeatedly mentioned as a main disadvantage of subcutaneously growing tumors. Indeed, staining for the endothelial marker CD31 [26] revealed a similar vascularisation in larger i.h. and s.c. grown HB. In the smaller i.h. nodules the blood supply seems to arise from the surrounding liver parenchyma, which shows a higher density of CD31 positive cells directly next to the tumor. There was no tendency in the i.h. tumors towards better vascularisation compared to s.c. tumors. Further immunohistological analysis of different markers of HB revealed few differences between i.h. and s.c. grown tumors.

The remarkable difference of orthotopic and ectopic growing tumor cells reported in literature and observed in our model is the distribution pattern of β-catenin [17]. β-catenin shows mutations in up to 80% of primary HB [27]. A nuclear accumulation of β-catenin was proposed as a useful marker of malignant paediatric liver tumors [28]. Translocated β-catenin in the nucleus acts as a transcription factor, whereas localization on the cell membrane correlates with the function as an adhesion molecule. β-catenin is involved in two apparently independent processes, cell-cell adhesion and signal transduction [29]. In the Wnt/β-catenin signaling pathway, Wnt proteins bind to receptors of the Frizzled and LRP families on the cell surface. Through several cytoplasmic relay components, the signal is transduced to β-catenin, which enters the nucleus and forms a complex with TCF to activate transcription of Wnt target genes [30]. The observation of β-catenin accumulation in the nucleus in orthotopically grown HB cells is an indicator of how the particular milieu influences the cell behavior, compared to s.c. grown HB cells with more cytoplasmic accumulated β-catenin. Thus an experimental model with i.h. HB may be more difficult to treat than a model with s.c. HB. However, this model more realistically reflects the situation of primary high risk hepatoblastoma in children and seems to be an interesting tool for preclinical testing of drug efficiency. It also provides the possibility of further evaluation of proliferation, metastatic potential /invasiveness, and vascular supply of HB liver nodules. Evaluation of interaction between liver parenchyma and chemotherapeutic drugs with respect to the possible changes in the repertoire of surviving HB cells becomes possible. Immunotherapeutical approaches in the treatment of intrahepatic HB may be tested similar to the elegant murine HCC model of Belnoue [31]. The fact of splenectomy in our model may be a limitation of further studies in combination with hematopoietic stem cell engraftment as the spleen is one main important differentiation site [19]. But human hematopoiesis in humanized NSG mice could be detected not only in the bone marrow but particularly in the liver of these mice [32]. Detailed investigations of interactions between differentiated human immune cells in the context of an adoptive immune transfer to HB-transplanted NSG mice eventually even profit by splenectomy because of higher amounts of immune cells in the livers of these mice [33].

With this orthotopic HB xenograft, we have established a feasible and reliable experimental model. Distribution of β-catenin in the HB cells and their interaction with the liver matrix in this model more adequately mirrors HB in situ. Testing of new therapeutic strategies in this HB model may therefore lead to a better clinical outcome when translated into patients.

## Materials and Methods

### Cell Cultures

The human HB cell lines HuH6 and HepT1 were used for all experiments [5]–[7]. HepT1was derived from a multifocal embryonal HB. Tumor cells are polyploid with 65–125 chromosomes per cell presenting deletions on 1p and 11q as well as a 6q15 translocation. The HepT1 cell line was characterized by Pietsch et al. [5]. HUH6 was derived from a mixed HB with focal chondroosteogenic tissue and a squamous cell morphology. Tumor cells present 48 chromosomes per nucleus. The HUH6 cell line was characterized and published by Doi [6]. The HB cells express α-fetoprotein. The cells were transduced with a plasmid encoding Gaussia luciferase (GLuc) (pCMV-GLuc, NEB, Frankfurt am Main, Germany) using Hi PerFect (Quiagen). Stable clones were isolated and maintained in DMEM (GIBCO BRL, Carlsbad, CA) supplemented with 10% FCS and G418. Cell cultures were maintained in a humidified atmosphere containing 5% CO_2_ at 37°C. Subconfluent cultures of HuH6 and HepT1 were trypsinized, suspended at 5×10^4^ cells/µl in medium without FCS for injection. All cells were mycoplasma negative.

### Animal experiments

NOD.Cg-Prkdcscid IL2rgtmWjl/Sz (NSG) mice were purchased from Charles River (Sulzfeld, Germany) and bred in our facility. HB cells were injected inboth male or female 4- to 6-week-old mice (24–30 g), kept in filter-top cages at 22°C, 60% humidity. Sterilized food and water were accessible ad lib.

### Ethics

This study was carried out in strict accordance with the recommendations in the Guide for the Care and Use of Laboratory Animals of the National Institutes of Health. The protocol was approved by the the responsible animal welfare authority of the University Tuebingen, the German Ethical Committee (Regional Counsil, approval ID: CK1/09). All surgery was performed under anesthesia, and all efforts were made to minimize suffering.

### Injection of tumor cells

For i.v. injection mice were exposed to red light for 5 minutes and then received 10^6^ cells in the tail vein. Another group received 10^6^ HB cells via intraperitoneal (i.p.) injection. Subcutaneous xenotransplantation of HB cells was performed as described previously [9].

For intrasplenic (i.s.) injections and splenectomy mice were anesthetized i.p. (xylazin 8 mg/kg, and ketamin 100 mg/kg body weight). The spleen was exposed via a left lateral laparotomy. 1×10^6^ HB cells were injected into the spleen. Splenectomy was carried out using high temperature battery-cautery (Faromed, Berlin) two minutes after cell injection. The incision was sutured with 5/0 monofilament absorbable synthetic sutures. Mice were given a subcutaneous injection of 5 mg/kg caprofen for analgesia. Postoperatively, mice were kept warm and returned to their cages when fully awake.

Blood samples were taken weekly from the retro-orbital plexus in CO_2_/O_2_ – anaesthetized mice. Mice were sacrificed when AFP and GLuc levels were increasing, when s.c. tumors reached a diameter of 20 mm, or when mice lost more the 10% of their body weight. Intraperitoneal organs and lungs were exposed and macroscopically assessed for existence of tumor. A portion of each tumor was fixed in 4% buffered formaldehyde and processed for histological analysis, while another portion was frozen in liquid nitrogen and stored at −80°C.

### Serum proteins

Human α-Fetoprotein (AFP) was measured in serum using an ELISA Kit (DRG Instruments, Marburg, Germany) and expressed in IU/mL. The analytic sensivity was found to be 1.78 IU/mL. GLuc activity as relative light units per second (RLU/s) was measured as described elsewhere (13). The background activity of GLuc was 12.000 RLU.

### Magnetic Resonance Imaging

Starting three weeks post operation weekly Magnetic Resonance Imaging (MRI) was carried out to determine the localization and extent of HB in the mice. Images were obtained with a 3 Tesla MRI clinical scanner (Magnetom Trio, Siemens, Erlangen, Germany). Three anaesthetized mice (Xylazin and Ketamin) were analyzed simultaneously in the prone position. Based on these images, transversal and coronar T1-/T2-weighted scans and transversal fast imaging with steady precession (TrueFISP) were obtained. A short localizer sequence was performed in order to effectively assess the following sequences. Transverse T1 weighted spinecho sequence: FOV 10 cm; data matrix 448, 15 slices; TR/TE 400/13 ms, flip angle 90°, slice thickness 1.5 mm, in-plane resolution 0.3×0.3 mm2, scan time 6 min. coronal T2-weighted turbo-spin echo sequence: FOV 11 cm, data matrix 348, 15 slices, TR/TE 3800/42 ms, flip angle 150°, slice thickness 1.5 mm, in-plane resolution 0.3×0.3, scan time 6,4 min. transverse T2-weighted turbo-spin echo sequence: FOV 9 cm, data matrix 448, 15 slices, TR/TE 3800/89, flip angle 150°, slice thickness 1,5 mm, in-plane resolution 0.2×0.2, Fat saturation, scan time 9,24 min., coronal TrueFisp: FOV 12,8 mm,data matrix 348, TR/TE 7.42/3.71 ms, flip angle 70°, slice thickness 1,5, in-plane resolution 0.3×0.3, scan time 1,28 min.

### Histology and Immunohistochemistry

Tumor specimen were fixed in formalin (37%). Tissue processing was continued in a vacuum tissue processor (Leica TP 1050, Leica Wiesloch, Germany). Sections of 3 µm were stained with hematoxylin/eosin. Immunhistochemistry in paraffin sections was performed using the ABC method as described previously [9]. Frozen specimens were sectioned into 7 µm sections using a Leica Cryotom. Sections were fixed with methanol/acetone 1∶1 at −20°C. To block unspecific binding areas slices were incubated in goat-serum (1%, DAKO, Hamburg, Germany). The following antibodies were used: CD31 (vascularity), Ki-67 (proliferation), AFP; Vimentin, Cytoceratin 7 (CK7), Cytoceratin AE (CK-AE), CD326 (tumor proteins); β-Catenin and E-cadherin (Wnt-β-Catenin signaling).The antibodies and the specific conditions used are described in Table 4. Nuclei were counterstained with DAPI (4′.6-Diamidino-2-phenylindole dihydrochloride, 0.1 ng/ml, Sigma, Munich, Germany). Immunofluorescence microscopy was carried out on a Zeiss Axio Scope epifluorescence microscope (Carl Zeiss, Oberkochen, Germany) with an MRC5 camera. Confocal-style three-dimensional imaging was performed using an Axio imager 2 microscope with ApoTome system (Carl Zeiss, Jena, Germany). Images were processed using AxioVision 4.8.1 software.

**Table 4:** Description of the used antibodies for immuno-histological staining.

Antigen	Host	Clone	Distributor	Dilution	Tissue specimen	Secondary Antibody
CD31	Rat	S231	Dianova	1∶10	Paraffin	2
AFP - HRP	Goat	C-19	Santa Cruz	1∶100	Paraffin	2
Ki-67	Mouse	MIB-1	DAKO	1∶75	Paraffin	2
Vimentin	Mouse	Vim3b4	DAKO	1∶100	Cryo	1
Cytokeratin 7	Mouse	OVTL12/30	DAKO	1∶100	Cryo	1
Cytokeratin	Mouse	AE1/AE3	DAKO	1∶100	Cryo	1
CD326-PE	Human	HEA-125	Miltenyi Biotec	1∶100	Cryo	-
ß-catenin – FITC	Mouse	14/B-catenin	BD	1∶100	Cryo	-
E-cadherin	Mouse	67A4	AG Bühring	1∶5	Cryo	1
CD133	Mouse	W6B3H10	AG Bühring	1∶5	Cryo	1

1: Alexa Fluor 546 goat anti-mouse IgG PE (1∶500).

2: Biotin-labeled Rabbit anti-rat (1∶100).
